# Successful Multidisciplinary Management of a Staple-Line Leak Following Laparoscopic Sleeve Gastrectomy Using Endoscopic Mega-stenting and Interventional Radiology-Guided Drainage: A Case Report

**DOI:** 10.7759/cureus.112997

**Published:** 2026-07-20

**Authors:** Mohammed Hamad Alrashedi

**Affiliations:** 1 General Surgery, King Fahad Specialist Hospital, Tabuk, SAU

**Keywords:** bariatric surgery complications, endoscopic mega-stent, laparoscopic sleeve gastrectomy, percutaneous drainage, staple-line leak

## Abstract

Staple-line leak (SLL) following laparoscopic sleeve gastrectomy (LSG) carries significant morbidity and, when diagnosis is delayed, can be life-threatening, occurring in a small but clinically important proportion of patients undergoing the procedure. Despite advances in bariatric surgery, management of post-LSG leaks remains challenging and often requires a multidisciplinary approach involving surgery, interventional radiology (IR), gastroenterology, and infectious disease specialists.

We report a 39-year-old medically free male who presented to our emergency department 10 days following LSG performed in Egypt, with a three-day history of progressive epigastric pain, nausea, vomiting, and fever. Imaging revealed an 18 × 12.5 × 12 cm perigastric collection with an air-fluid level consistent with a staple-line leak. The patient was managed in the ICU with broad-spectrum antibiotics, bowel rest, and percutaneous pigtail drainage. A follow-up CT scan confirmed active oral contrast extravasation from the superior residual stomach. Due to the unavailability of an endoscopic mega-stent at our institution, the patient was transferred to an advanced bariatric center, where successful endoscopic mega-stent insertion was performed on postoperative day 19. The stent was removed 78 days later without complications.

At the eight-month follow-up, the patient was asymptomatic, with a weight of 68 kg (BMI 23.85 kg/m²), representing approximately 86% excess weight loss, an excellent functional and metabolic outcome.

This case highlights the critical importance of early radiologic diagnosis, prompt percutaneous drainage, stepwise escalation of antibiotic therapy, and timely access to advanced endoscopic intervention in managing post-LSG staple-line leaks. Institutional capacity to deploy endoscopic mega-stents and established interhospital referral pathways are key determinants of outcome.

## Introduction

Laparoscopic sleeve gastrectomy (LSG) has established itself as the most commonly performed bariatric operation worldwide. According to the most recent IFSO Worldwide Survey (2020-2021), sleeve gastrectomy remained the dominant bariatric procedure globally, with nearly 600,000 metabolic and bariatric surgery (MBS) procedures performed in 2021 alone [1]. Updated guidelines from the American Society for Metabolic and Bariatric Surgery (ASMBS) and IFSO (2022) further expanded surgical eligibility criteria, underscoring the growing reach of LSG as a therapeutic modality for obesity and its metabolic comorbidities [2]. Its appeal lies in technical reproducibility, the absence of intestinal anastomosis, and well-characterized long-term weight outcomes [3]. Nevertheless, LSG carries procedure-specific risks, of which staple-line leak (SLL) represents the most feared and potentially lethal complication.

The reported incidence of post-LSG SLL ranges from 0.7% to 5% in contemporary series, with the majority occurring within the first 10 days after surgery [4]. Leaks most commonly originate at the proximal staple line near the gastroesophageal junction, an anatomic region characterized by relative ischemia, higher intraluminal pressure, and tissue tension, factors described extensively in recent reviews of SLL pathogenesis [5]. The clinical presentation is frequently nonspecific, with tachycardia as the most sensitive early sign. A missed or delayed diagnosis can result in frank peritonitis, septic shock, and a substantially increased mortality risk [6]. A 2024 review of updated literature on leak risk factors confirmed that technical variables, including bougie size, antrum resection extent, and staple-line reinforcement technique, remain important, though no single technical modification has been proven to eliminate SLL entirely [5].

Managing post-LSG SLL has changed considerably over the last decade, and not in a single direction. It has been a gradual, sometimes messy, shift driven by accumulated institutional experience rather than any single landmark trial. The broad direction has been away from early operative re-exploration and toward a more conservative, step-by-step approach. Drain the collection, rest the gut, get on top of the infection, then deal with the defect endoscopically. Surgery is reserved for patients who are too unstable to wait or those who present with frank peritonitis [7].

The endoscopic data are now fairly solid. A review covering 40 studies and over 490 patients found closure rates around 92%, a number that, at first glance, seems almost too good but holds up reasonably well across different centers and techniques [8]. What has become clearer more recently is that stent size actually matters enormously. Standard-size stents largely fail in this context, whereas esophagoduodenal mega-stents achieved closure in around 80% of cases [9], a difference large enough that there is little point in placing anything smaller. Compared with other available endoscopic approaches, including over-the-scope clips (OTSC), endoscopic internal drainage (EID), and endoscopic vacuum therapy (EVT), mega-stents remain the most widely adopted modality for post-LSG SLL, owing to their ability to bridge larger defects and sustain luminal diversion over extended dwell periods without requiring repeat endoscopic access [8,10].

Where things get complicated in practice is access to equipment. Not every bariatric program has mega-stents on the shelf, and not every center has a gastroenterologist experienced enough to deploy them. That gap is not trivial. When the interval between identifying a leak and achieving definitive stenting stretches from days into weeks, patients accumulate ICU time, total parenteral nutrition (TPN)-related complications, and infectious risk that are entirely avoidable. This is not a theoretical concern; it played out directly in the case described here. The recent publication of a comprehensive narrative review on managing leaks and fistulas after LSG (2025) further highlights that no universally agreed-upon algorithm exists and that institutional capability gaps continue to shape clinical decision-making [10].

We present a case of post-LSG SLL managed via a stepwise multidisciplinary protocol encompassing emergency CT-guided diagnosis, ICU-level supportive care, interventional radiology (IR)-guided pigtail drainage, infectious disease-directed antibiotic escalation, TPN, and ultimately successful endoscopic mega-stent insertion following interhospital transfer to a referral center. The case illustrates both optimal management principles and the real-world logistical challenges that clinicians face when critical technology is unavailable at the treating institution.

This case has not been previously presented at any conference, symposium, or scientific meeting, and no part of this work has been published in abstract or poster form.

## Case presentation

Patient demographics and background

The patient was a 39-year-old medically free male of Egyptian nationality, 169 cm tall, weighing 107 kg at presentation (BMI 37.5 kg/m²). There was no known chronic medical history. The primary complaint was epigastric pain, nausea, vomiting, and fever commencing three days prior to presentation, ten days following laparoscopic sleeve gastrectomy performed in Egypt. At final follow-up, weight had decreased to 68 kg with a BMI of 23.85 kg/m², representing approximately 86% excess weight loss.

Chief complaint and history of presenting illness

The patient had been experiencing epigastric pain for about three days before presentation. The pain was sharp, localized, non-radiating, and unresponsive to analgesics taken at home. It was aggravated by eating. He also reported persistent nausea, repeated vomiting, and fever, although he had not measured his temperature before presentation. There was no history of hematemesis, melaena, or shoulder tip pain. He denied any change in bowel habits or urinary symptoms.

The review of systems was otherwise unremarkable. The patient had not received antibiotic therapy before presentation and had been managed conservatively at home following discharge from the surgical facility in Egypt.

Physical examination

On physical examination, the patient appeared generally unwell but was hemodynamically responsive. He was not clinically jaundiced and had no pallor on conjunctival examination.

On arrival, blood pressure was 130/82 mmHg, oxygen saturation on room air was 98%, heart rate was 115 bpm, and temperature was 37.9°C, neither dramatically abnormal in isolation, but together concerning in someone 10 days out from abdominal surgery. General appearance was of an unwell patient, not jaundiced, not pale. Chest examination revealed equal bilateral air entry. Abdominal palpation found a soft, lax abdomen with localized tenderness over the epigastric region and left upper quadrant, without rigidity or generalized peritonism.

Investigations

Laboratory Findings

Initial hematology demonstrated a leukocytosis (WBC 13×10³/µL) and mild anemia (Hgb 11.0 g/dL). Serum potassium was mildly low at 3.0 mmol/L. Serial laboratory values over the admission period are presented in Table 1.

**Table 1:** Serial laboratory results over the course of admission WBC: white blood cell count, CRP: C-reactive protein, ESR: erythrocyte sedimentation rate.

Parameter	Day 1	Day 2	Day 3	Day 7	Reference values
WBC (×10³/µL)	13.0	15.0	11.0	9.0	4.5–11.0 × 10³/µL
Hemoglobin (g/dL)	11.0	10.5	10.0	11.7	13.5–17.5 g/dL (male)
CRP (mg/L)	—	70	—	—	<5 mg/L
ESR (mm/hr)	—	50	—	—	0-15 mm/hr (male)

The WBC actually climbed to 15 on Day 2, which was not unexpected. The collection was still there, the infection was still active, and source control had not yet been achieved. The CRP came back at 70 and ESR at 50, both consistent with what would be expected at that stage. Once the pigtail was in and the antibiotics were upgraded, things started moving in the right direction. Inflammatory markers trended down, the patient felt better, and clinically, it was clear the drainage had done its job.

Radiological Investigations

**Figure 1:**
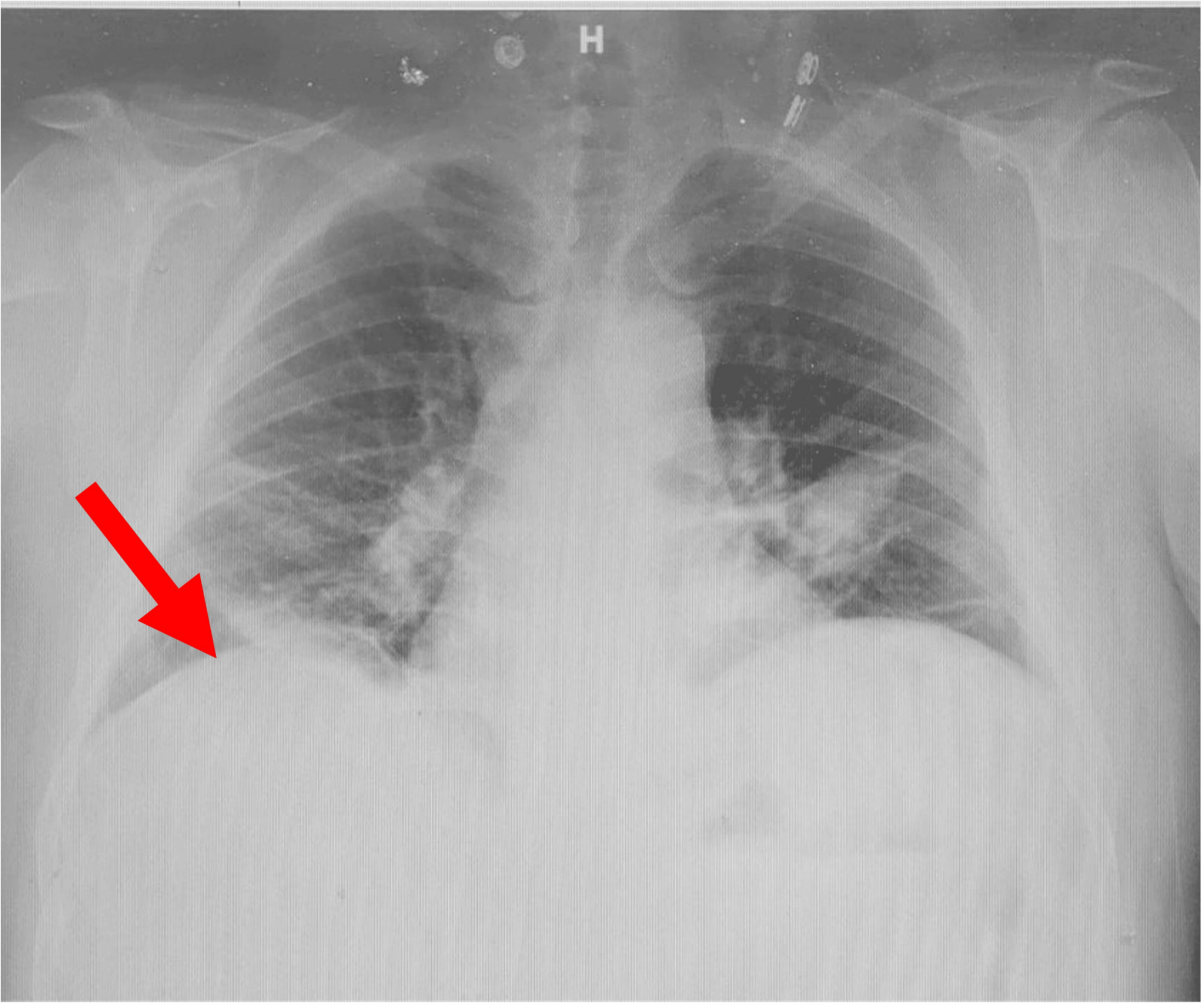
Chest X-ray at admission Admission chest radiograph demonstrating no free subdiaphragmatic air and clear lung fields bilaterally, excluding pneumoperitoneum.

A chest radiograph performed on admission excluded pneumoperitoneum, with no free subdiaphragmatic air identified, and the lung fields appeared clear (Figure 1). Computed tomography (CT) of the abdomen and pelvis with intravenous and oral contrast served as the cornerstone of diagnosis and follow-up. Radiological findings are summarized in Table 2. 

**Table 2:** Radiological investigations and findings

Investigation / Date	Modality	Findings	Clinical Significance
Chest X-Ray (Admission - Day 0)	PA chest radiograph	No air under diaphragm; lungs clear	Excluded hollow viscus perforation; no pneumoperitoneum
CT Abdomen with IV + Oral Contrast (Day 0)	Triple-phase CT with oral contrast	Large peri-gastric collection 18×12.5×12 cm at staple line; air-fluid level; blurred surrounding fat stranding	Consistent with post-LSG staple-line leak vs. infected hematoma
CT Abdomen with IV + Oral Contrast (Day 5)	Follow-up CT with oral contrast	Oral contrast extravasating into peri-gastric collection from superior residual stomach; decreased collection size; pigtail tip visualized at inferior collection	Confirmed active staple-line leak; adequate pigtail positioning
Upper Endoscopy (Day 19)	Flexible upper GI endoscopy	Visible gastric staple-line defect; mega-stent deployed successfully	Leak confirmed endoscopically; stent closure achieved

**Figure 2:**
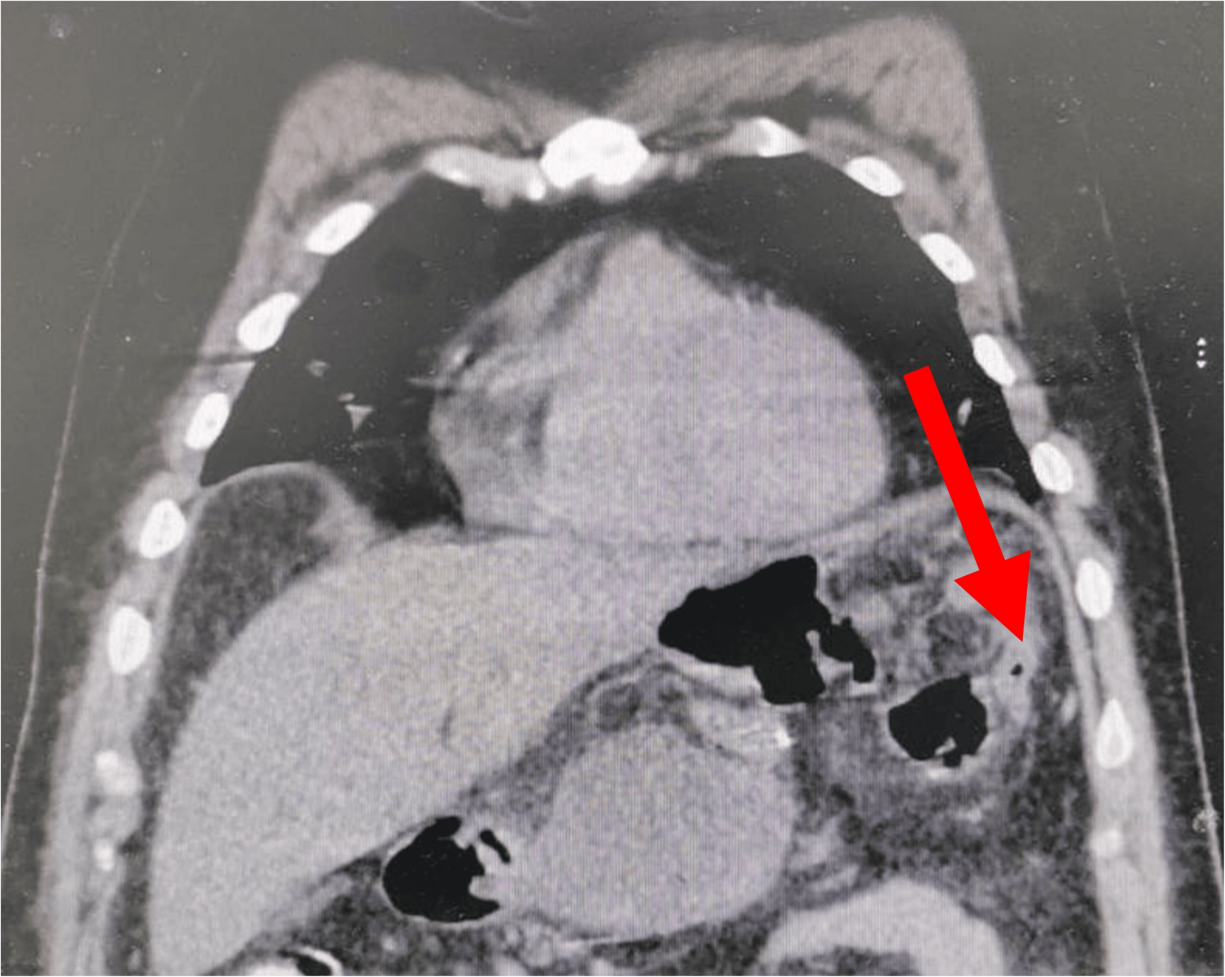
CT scan showing collection Axial CT abdomen with IV and oral contrast (Day 0) demonstrating a large peri-gastric collection at the staple line with an internal air-fluid level and surrounding fat stranding.

The first CT findings were about as clear as they could be. A collection measuring 18 × 12.5 × 12 cm was seen at the operative bed, with an air-fluid level visible inside it and surrounding fat stranding (Figure 2). The radiologist's impression was either a staple-line leak or an infected hematoma. Both were plausible at that point, and honestly, the distinction mattered less than getting a drain in. The follow-up CT five days later settled it. Oral contrast was clearly tracking into the perigastric space from the upper part of the residual stomach, which confirmed an active leak. On the positive side, the collection had shrunk somewhat compared with the first scan, and the pigtail was sitting where it needed to be. 

Clinical timeline and in-hospital management

The patient's clinical course is laid out in Tables 3, 4. In broad strokes, the approach was fairly straightforward. The patient was stabilized, the infection was brought under control, nutrition was provided intravenously, and the leak was managed definitively after transfer to a center that could provide the required care. 

**Table 3:** Pharmacological and supportive management summary ID: infectious diseases, IV: intravenous, BID: twice daily, TID: three times daily, QID: four times daily, OD: once daily, SC: subcutaneous, DVT: deep vein thrombosis, PE: pulmonary embolism, NS: normal saline, KCl: potassium chloride, TPN: total parenteral nutrition, NPO: nil per os, GI: gastrointestinal.

Drug / Intervention	Rationale	Duration	Notes
Ciprofloxacin 400 mg IV BID	Broad-spectrum antibiotics (empiric)	Days 0–1 (discontinued after ID consultation)	Discontinued after ID consultation
Metronidazole 500 mg IV TID	Anaerobic coverage (empiric)	Days 0–1 (discontinued after ID consultation)	Discontinued after ID consultation
Meropenem 1 g IV TID	Upgrade to carbapenem per ID	Day 1 onward	Escalated for polymicrobial/resistant coverage
Metoclopramide 10 mg IV TID	Antiemetic/prokinetic	Day 0 onward	Symptom control
Paracetamol 1 g IV QID	Analgesia/antipyretic	Day 0 onward	Fever management
Omeprazole 40 mg IV OD	Proton pump inhibitor	Day 0 onward	Mucosal protection
Enoxaparin 60 mg SC OD	Low-molecular-weight heparin	Day 0 onward	DVT/PE prophylaxis
D5 1/2 NS 150 mL/hr + KCl 20 mEq/L	IV fluid resuscitation + electrolyte replacement	Day 0 onward (reduced once TPN commenced)	Reduced to 80 mL/hr once TPN started
TPN 40 mL/hr	Total parenteral nutrition	Day 5 onward	Initiated after leak confirmation
NPO	Nil per os	Day 0 until stent insertion (Day 19)	GI rest to promote leak healing

**Table 4:** Chronological clinical timeline and disease progression ICU: intensive care unit, CT: computed tomography, NPO: nil per os, IV: intravenous, IR: interventional radiology, mL: milliliter, CRP: C-reactive protein, ESR: erythrocyte sedimentation rate, HR: heart rate, WBC: white blood cell count, TPN: total parenteral nutrition, BMI: body mass index, EWL: excess weight loss.

Date	Event	Details
Day 0 (Admission)	ICU admission	Emergency presentation; CT showed 18 × 12.5 × 12 cm peri-gastric collection with air-fluid level; admitted to ICU; empiric antibiotics, NPO, IV fluids initiated
Day 1	IR drainage	Pigtail drain inserted by interventional radiology; 200–300 mL turbid infected hematoma drained; CRP 70, ESR 50; antibiotics escalated to meropenem
Day 2	Clinical improvement	Clinically improving; afebrile (36.8°C); HR normalizing to 105; drain output 100–150 mL; WBC trending down to 11
Day 5	Leak confirmed on CT	Follow-up CT confirmed active oral contrast extravasation from the superior residual stomach; TPN initiated; gastroenterology consulted for endoscopic stenting
Day 6	Stent unavailable	Mega-stent not available at treating institution; decision made to transfer to advanced bariatric center; referral submitted
Day 15	Awaiting transfer	Patient stabilized in ICU; continued NPO + TPN; referral acceptance pending
Day 19	Endoscopic stenting	Upper endoscopy performed at receiving hospital; visible staple-line defect confirmed; mega-stent inserted without complications
Day 29	Discharge	Discharged from the private hospital in good condition after 10 days post-stenting
Day 46	Outpatient follow-up	Reviewed in bariatric clinic; asymptomatic; stent in situ
Day 80	Stent removal	Successful endoscopic stent removal at an advanced bariatric center; discharged the same day
Day ~256	Final follow-Up	Fully asymptomatic; weight 68 kg, BMI 23.85 kg/m² (EWL ~86%); excellent functional outcome

ICU Admission and Initial Stabilization (Days 1 and 2)

Upon admission, the patient was transferred directly to the intensive care unit for hemodynamic monitoring and initiation of management. The patient was kept nil by mouth from the outset. There was no real debate about that, given the circumstances. Fluids were administered at 150 mL/hr, consisting of 5% dextrose in half-normal saline with potassium added to the bag, since his potassium on admission was 3. Antibiotics, ciprofloxacin and metronidazole, were started empirically. Reasonable coverage for the likely organisms was provided while culture results were pending. Metoclopramide was administered for nausea, paracetamol for fever, omeprazole, and enoxaparin for clot prophylaxis.

Interventional Radiology Drainage (Day 2)

With the CRP at 70, ESR at 50, and that collection sitting there on CT, waiting was not a reasonable option. Interventional radiology was consulted, and a pigtail catheter was placed under CT fluoroscopic guidance the same day. The procedure was straightforward, with no complications. Drainage yielded somewhere between 200 and 300 mL of turbid, infected material, consistent with an infected hematoma rather than a simple reactive collection. Microbiological samples were obtained from the aspirate for culture and sensitivity. Infectious disease was formally consulted, and antibiotic therapy was escalated to meropenem 1 g IV three times daily, with discontinuation of ciprofloxacin and metronidazole. Septic screening was initiated.

Clinical Stabilization (Days 3-5)

Over the subsequent 48 hours, the patient demonstrated steady clinical improvement. He became afebrile (36.8°C), with progressive normalization of heart rate (105 bpm) and resolution of abdominal tenderness. The WBC count decreased to 11 × 10³/µL, and the daily drain output decreased to 100-150 mL. No new septic focus was identified on microbiological screening.

Leak Confirmation and Nutritional Optimization (Day 6)

**Figure 3:**
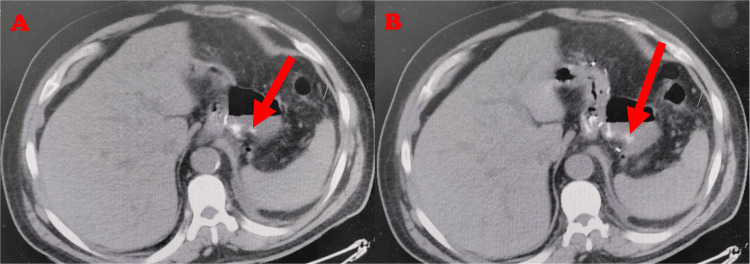
CT contrast extravasation Follow-up CT abdomen with oral and IV contrast (Day 5). (A) Axial CT demonstrating a large peri-gastric collection at the operative bed with an internal air-fluid level. The red arrow points to the air-fluid level within the collection at the staple-line site. (B) Consecutive axial slice demonstrating active oral contrast extravasation from the superior aspect of the residual stomach into the peri-gastric space. The red arrow highlights the point of active contrast leakage, confirming a communicating staple-line leak.

The Day 5 CT settled any remaining uncertainty. Oral contrast was clearly tracking into the perigastric collection from the upper part of the residual stomach. This confirmed the presence of an active leak (Figures 3A and 3B). The collection itself had actually shrunk a little relative to the admission scan, which was reassuring, but the extravasation meant conservative monitoring was no longer sufficient. TPN was started at 40 mL/hr. This was sufficient to cover caloric and protein needs without putting anything through the gut. Intravenous fluid administration was correspondingly reduced. Gastroenterology was consulted for consideration of endoscopic mega-stent insertion.

Inter-hospital Transfer and Endoscopic Stenting (Days 7-20)

**Figure 4:**
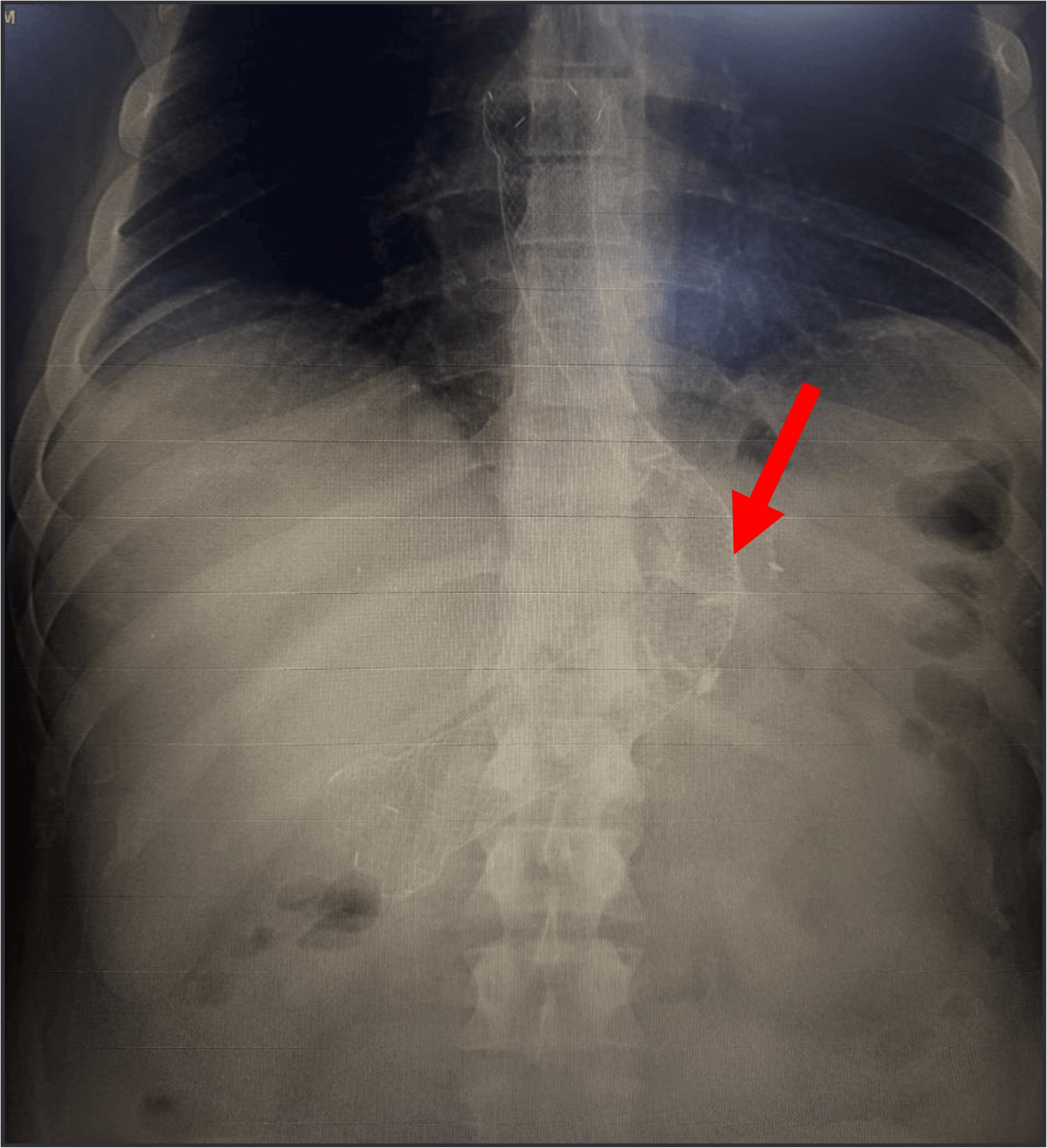
X-ray post stent insertion Plain abdominal radiograph following endoscopic mega-stent insertion (Day 19) confirming stent positioning across the staple-line defect.

Once the leak was confirmed on CT, the next step was clear. Endoscopic mega-stent insertion was indicated. The problem was that the device was simply not available at the treating institution. A referral was sent to an advanced bariatric center with the necessary endoscopic setup, and then came the waiting. The patient remained clinically stable, with no acute deterioration, but stayed in the ICU on NPO and TPN until the referral was completed. Transfer eventually occurred on Day 16, and endoscopy was performed at the receiving center on Day 20, postoperative day 19. The defect was visible on endoscopy, and the mega-stent was inserted without any issues (Figure 4). The patient was discharged from the referral center approximately 10 days after stent placement. 

Stent Removal and Follow-Up (November 2025 to April 2026)

At the outpatient bariatric clinic review on Day 47, the stent remained in situ, and there were no complaints. Stent removal was planned after an adequate dwell time to ensure leak healing. Stent removal went ahead on Day 81 at the Advanced Bariatric Center. The procedure was straightforward, performed as a same-day procedure, and without complications. At the most recent clinic review in late April 2026, roughly eight months after the leak was first diagnosed, there were no complaints. Weight had decreased to 68 kg, with a BMI of 23.85, representing about 86% excess weight loss from baseline. By any measure, an excellent outcome.

Outcomes

The total ICU stay spanned approximately 15 days. Combined hospitalization across both the treating and referral institutions totaled roughly 25 days. The mega-stent remained in situ for approximately 78 days before elective endoscopic removal, with the entire episode from leak diagnosis to stent removal spanning around 80 days. No complications directly attributable to the management interventions were encountered, and 30-day mortality was nil. At final follow-up, approximately eight months after the index presentation, weight had decreased from 107 kg to 68 kg, with a BMI of 23.85 kg/m², firmly within the normal range, representing approximately 86% excess weight loss. The patient had returned to a normal diet and reported no ongoing symptoms. Notably, a final BMI of 23.85 kg/m² places the patient firmly within the normal weight category per the World Health Organization classification. This was a remarkable outcome given a presenting BMI of 37.5 kg/m² and underscores that the complication, though serious, did not preclude the intended bariatric benefit of the procedure.

## Discussion

Epidemiology and risk factors of post-LSG staple-line leak

Staple-line leak following LSG occurs in 0.7-5% of contemporary series, with the majority occurring within the first 10 postoperative days. Although the absolute risk is low, the disproportionate severity of SLL relative to its frequency, with reported mortality as high as 3.7%, justifies heightened vigilance in the postoperative period [9]. A single-center retrospective cohort of 402 patients confirmed that percutaneous drainage and endoscopic stenting remain the two most commonly used management modalities, with operative reintervention required in fewer than 5% of cases [4]. Recognized patient-level risk factors include male sex, BMI exceeding 50 kg/m², previous gastric surgery, and smoking. These factors intersect with the technical determinants of SLL, including bougie size, extent of antrum resection, proximity to the angle of His, and staple-line reinforcement strategy [5].

The timing of presentation in the current case, 10 days postoperatively with symptoms commencing approximately 3 days prior, is consistent with the Type II/Type III boundary in the Rosenthal classification, which categorizes leaks by temporal onset [11]. Localization near the superior staple line is characteristic and reflects the pathophysiological triad of relative ischemia, mechanical tension, and distal functional obstruction that increases intraluminal pressure at the proximal sleeve. A study from a high-volume regional referral center in Qatar, directly relevant to this GCC-based case, found that tachycardia was present in over 85% of patients at the time of leak diagnosis, confirming its primacy as an early warning sign [6].

Diagnostic approach

CT abdomen with IV and oral contrast remains the gold standard imaging modality for the diagnosis of post-LSG SLL, with sensitivity exceeding 90% when findings of oral contrast extravasation, perigastric collections, or fat stranding are used as diagnostic criteria [12]. A study further highlighted the variable radiological presentation of SLL and the need for a high index of clinical suspicion even when early CT findings are equivocal [6]. That scan alone was enough to make the decision: ICU, source control, no delay.

The Day 5 follow-up CT mattered just as much, arguably more. Conservative management made sense while the drain was in and the inflammatory markers were improving, but the moment contrast was seen tracking into the perigastric space from the upper stomach, the plan had to change. Serial imaging in this setting is not just a box-ticking exercise. It is genuinely how management decisions get made, and the evidence supports building it into the protocol rather than treating it as optional [7]. A 2024 update on staple-line leak risk factors and management standards reinforces the importance of structured radiological follow-up in bariatric programs [5].

Nonoperative management strategy

The management of post-LSG SLL has undergone a paradigm shift from operative reintervention toward a primarily non-operative, multidisciplinary, stepwise approach. This transition is supported by a substantial and growing body of evidence. A study from a large GCC bariatric program reported a 92% non-operative resolution rate in 73 consecutive SLL patients managed via IR drainage, endoscopic stenting, and antibiotic therapy, establishing a reproducible management algorithm applicable to the present case [7]. A comparable series published in Langenbeck's Archives of Surgery is worth noting here, not because it changes the overall picture, but because it adds some nuance. In stable patients, conservative management without stenting produced outcomes similar to those with stenting, a useful reminder that the intervention needs to fit the clinical situation rather than being applied uniformly [13].

The four pillars of non-operative SLL management, (1) source control via percutaneous drainage, (2) bowel rest and nutritional support, (3) antimicrobial therapy, and (4) definitive endoscopic closure, are fully reflected in the management of the present case. IR-guided pigtail catheter placement, providing immediate drainage of 200-300 mL of a turbid, infected hematoma, achieved rapid source control and reduced systemic septic burden. The subsequent inflammatory marker improvement (WBC 13 → 11 × 10³/µL; CRP 70 → improving) and clinical stabilization confirm that IR drainage effectively bridges patients to definitive therapy without the morbidity of operative exploration.

With regard to antimicrobial stewardship, the escalation from empiric ciprofloxacin/metronidazole to meropenem following ID consultation exemplifies current best practice principles. Managing leaks and fistulas after LSG, as comprehensively reviewed in a recent narrative review, requires culture-directed therapy given the polymicrobial nature of infected perigastric collections and the growing risk of resistant organisms in patients who have received prior antibiotics or have been managed abroad [10].

Role of endoscopic mega-stenting

Endoscopic placement of fully covered self-expanding metallic stents (FCSEMS), and specifically esophagoduodenal mega-stents, has become the cornerstone of definitive non-operative SLL management. The mega-stent diverts luminal content away from the staple-line defect, reduces intraluminal pressure, and creates a mechanical seal that facilitates tissue healing. The data on this point are hard to argue with. Success rates with mega-stents were around 80%, compared with roughly 25% with standard-size stents. The gap is wide enough that there is little justification for using anything smaller [9]. That finding sits at the center of what went wrong logistically in this case. The mega-stent was the right tool; it was not on the shelf, and that gap cost nearly two weeks of ICU time.

The broader stenting literature tells a consistent story. A review that pulled together 40 studies and close to 500 patients reported an overall closure rate of 92%, a strong number, though the 23% stent migration rate is a real caveat that does not always get the attention it deserves [8]. A more focused meta-analysis published in 2025, focusing specifically on post-sleeve leaks, reached similar conclusions [14], as did a 2022 review of the wider bariatric stenting literature [15]. Across all three, the picture is reasonably coherent: endoscopic stenting works in most cases, mega-stents work better than smaller ones, and migration remains the main thing to watch for.

Stent dwell time remains a subject of ongoing debate. Most institutions advocate 6-8 weeks before planned endoscopic removal, though some series have reported safe removal at 4 weeks in cases with early radiological evidence of healing. In the present case, a stent dwell time of approximately 78 days reflects a shared decision-making process influenced by the inter-institutional transfer pathway and the absence of evidence of residual leak before removal. Stent removal was uncomplicated. This outcome is consistent with large published series [9].

A critical systemic lesson from this case is the clinical consequence of mega-stent unavailability. The 9-day delay between stenting indication and actual stent deployment prolonged the patient's ICU stay, increased exposure to TPN-related risks, and extended total hospitalization. A 2021 case report from the National Guard Hospital (Al-Madinah, Saudi Arabia) similarly documented delayed endoscopic intervention due to stent unavailability, underscoring that this is a regional, not isolated, institutional challenge [16]. Bariatric accreditation bodies, including ASMBS and IFSO, should consider mandatory access to mega-stenting capability, or documented, operationally tested inter-hospital referral pathways, as a minimum quality standard for certified bariatric programs. The establishment of regionalized care pathways for complex bariatric complications, analogous to trauma network models, represents a practical and achievable systems-level intervention that could meaningfully reduce the interval between leak diagnosis and definitive endoscopic therapy [10,17].

The multidisciplinary team in bariatric complications

This case epitomizes the centrality of multidisciplinary team (MDT) coordination in achieving successful outcomes in the management of post-bariatric complications. Getting a good outcome in a case like this does not depend on one team or one intervention. It comes down to everyone being involved at the right time. Emergency medicine flagged it early, surgery directed the overall plan, interventional radiology dealt with the collection, infectious disease rationalized the antibiotics, gastroenterology handled the stenting decision, intensive care kept things from deteriorating in the meantime, and nutrition kept the metabolic situation from becoming its own problem. A review makes this point clearly: what distinguishes good outcomes in bariatric complications is not simply having specialists available, but having them engaged together rather than in sequence [17].

A recent narrative review on managing leaks and fistulas after LSG reinforces that clinical pathways integrating bariatric surgery, endoscopy, and interventional radiology, with pre-established inter-hospital referral protocols, are the hallmark of high-performing bariatric programs [10]. Resource allocation decisions, particularly regarding endoscopic equipment procurement and specialist staffing, should be informed by anticipated complication rates in high-volume LSG programs.

Nutritional considerations

Total parenteral nutrition (TPN) is the most widely utilized modality for nutritional support in post-LSG SLL, given the requirement for complete gastrointestinal rest. In the present case, TPN was initiated at 40 mL/hr following CT confirmation of an active leak on Day 5 of admission. A management series reported that patients managed with enteral feeding jejunostomy achieved a significantly shorter time to leak resolution than those receiving TPN (HR = 0.5, p = 0.047), suggesting that jejunal feeding, when anatomically feasible and technically achievable, may offer metabolic advantages over parenteral nutrition [7]. Clinicians should individualize the nutritional approach based on patient stability, leak anatomy, available resources, and the expected duration of bowel rest.

Long-term bariatric outcome

A clinically significant finding in this case is the exceptionally long-term bariatric outcome achieved despite a severe early complication. A final BMI of 23.85 kg/m² at the eight-month follow-up, representing approximately 86% excess weight loss, exceeds median outcomes reported for uncomplicated LSG in most registry-based series at 12 months [3]. This observation is consistent with a 2024 retrospective analysis, the largest study to date evaluating long-term outcomes specifically in post-LSG SLL patients, which demonstrated that patients who achieve leak resolution without operative reintervention achieve weight-loss outcomes comparable to those without SLL [18]. The same study confirmed that operative re-intervention (required in 27.8% of their cohort) was the key determinant of adverse long-term bariatric outcomes, further reinforcing the imperative of non-operative management success.

These findings carry an important prognostic message for patients experiencing post-LSG SLL: a serious early complication, when managed appropriately, need not compromise the intended metabolic benefit of the procedure. This information is valuable in patient counseling and shared decision-making, particularly in high-volume bariatric programs in regions such as Egypt and the UAE, where LSG volumes are rapidly growing [19].

Comparison with published literature and regional relevance

Internationally, the evolution toward non-operative management of post-LSG SLL is well supported. Two retrospective studies arrived at broadly the same conclusion from different datasets. Percutaneous drainage followed by endoscopic stenting has become the default pathway in most centers managing post-LSG leaks, and the outcomes data behind it are now fairly well established [4,20]. A review provided a framework for treatment selection based on leak chronicity and collection size. This approach closely mirrors the stepwise algorithm followed in the present case [7].

The regional dimension of this case adds important contextual value. Egypt has seen significant growth in bariatric surgery volumes since 2021, with sleeve gastrectomy representing the dominant procedure and revisional surgery rates rising in parallel [19]. Postoperative complications in patients who travel internationally for bariatric surgery, so-called "medical tourists," present unique challenges related to incomplete operative records, variable preoperative workup standards, and logistical barriers to follow-up [13]. This case demonstrates that systematic, protocol-driven management by an MDT at the receiving center can achieve excellent outcomes even in this complex patient subgroup.

Clinical and Institutional Lessons

Several practical lessons emerge from this case that are worth stating plainly. Tachycardia in any patient presenting within 30 days of LSG should trigger urgent CT evaluation. It is the most consistent early sign of a leak and should not be attributed to dehydration or anxiety without imaging to exclude an intra-abdominal source. CT with IV and oral contrast is the investigation of choice, and a single negative scan does not rule out a developing leak; serial imaging guided by clinical trajectory is the more defensible approach. Percutaneous pigtail drainage is not a temporizing measure of last resort. It is an active therapeutic intervention that achieves source control, reduces septic burden, and buys the time needed to plan definitive management safely. Antibiotic decisions benefit from early ID involvement, and the threshold for escalating to carbapenem coverage should be low in patients who have been managed abroad or received prior antibiotics. TPN should be initiated without delay once an active leak is confirmed. The gut needs to be at rest, and nutritional deficit accumulates quickly in this setting. Finally, and perhaps most importantly for program directors and institutional leads, the availability of endoscopic mega-stents is not a nice-to-have. It is a functional requirement for any bariatric program managing SLL. Where on-site capability does not exist, a documented, tested referral pathway to a center that can deliver this intervention within 24-48 hours of the decision being made is the minimum acceptable standard.

## Conclusions

This case documents the successful non-operative management of a post-laparoscopic sleeve gastrectomy staple-line leak through a coordinated multidisciplinary protocol. Early CT diagnosis, prompt percutaneous drainage, culture-directed antibiotic escalation, and total parenteral nutrition stabilized a critically unwell patient and created the conditions for definitive repair. Endoscopic mega-stent insertion, achieved following inter-hospital transfer due to device unavailability at the treating institution, resulted in complete leak closure without operative re-intervention. At the eight-month follow-up, the patient was asymptomatic with a BMI of 23.85 kg/m² and approximately 86% excess weight loss, demonstrating that a serious perioperative complication, when managed systematically, need not compromise the intended metabolic benefit of the procedure. The broader implication of this case is institutional. Bariatric programs managing high volumes of sleeve gastrectomy patients must either maintain on-site access to endoscopic mega-stenting or have pre-established, operationally ready referral pathways to centers that do. The delay encountered here, nearly two weeks between stenting indication and stent deployment, was avoidable and carries lessons for program-level quality improvement. Beyond the individual case, the findings point to a systems-level imperative: the creation of regionalized referral networks for complex bariatric complications, ensuring that advanced endoscopic capability is accessible within a clinically acceptable timeframe regardless of the treating institution's resources. Structured multidisciplinary protocols and inter-institutional agreements, rather than ad hoc referrals, are the foundation upon which equitable bariatric complication management must be built.
